# Distance and destination of retail meat alter multidrug resistant contamination in the United States food system

**DOI:** 10.1038/s41598-023-48197-z

**Published:** 2023-11-29

**Authors:** Gabriel K. Innes, Andrew N. Patton, Keeve E. Nachman, Joan A. Casey, G. Sean Stapleton, Alison G. Abraham, Lance B. Price, Sara Y. Tartof, Meghan F. Davis

**Affiliations:** 1 Johns Hopkins Bloomberg School of Public Health, Baltimore, MD USA; 2 Yuma Center for Excellence in Desert Agriculture, Yuma, AZ USA; 3 University of San Francisco Geospatial Analysis Lab, San Francisco, CA USA; 4 Center for a Livable Future, Johns Hopkins Bloomberg School of Public Health, Baltimore, MD USA; 5 Risk Sciences and Public Policy Institute, Johns Hopkins Bloomberg School of Public Health, Baltimore, MD USA; 6 Department of Environmental & Occupational Health Sciences, University of Washington, Seattle, WA USA; 7 Department of Epidemiology, Colorado School of Public Health, Aurora, CO USA; 8 Department of Epidemiology, Johns Hopkins Bloomberg School of Public Health, Baltimore, MD USA; 9 Department of Ophthalmology, University of Colorado, Anschutz Medical Campus, Aurora, CO USA; 10 Milken Institute School of Public Health, George Washington University, Washington, DC USA; 11 Kaiser Permanente Southern California, Pasadena, CA USA; 12 Department of Health Systems Science, Kaiser Permanente Bernard J. Tyson School of Medicine, Pasadena, CA USA

**Keywords:** Antimicrobials, Antimicrobial resistance, Risk factors, Bacterial infection

## Abstract

Antibiotic-resistant infections are a global concern, especially those caused by multidrug-resistant (MDR) bacteria, defined as those resistant to more than three drug classes. The animal agriculture industry contributes to the antimicrobial resistant foodborne illness burden via contaminated retail meat. In the United States, retail meat is shipped across the country. Therefore, understanding geospatial factors that influence MDR bacterial contamination is vital to protect consumers and inform interventions. Using data available from the United States Food and Drug Administration’s National Antimicrobial Resistance Monitoring System (NARMS), we describe retail meat shipping distances using processor and retailer locations and investigated this distance as a risk factor for MDR bacteria meat contamination using log-binomial regression. Meat samples collected during 2012–2014 totaled 11,243, of which 4791 (42.61%) were contaminated with bacteria and 835 (17.43%) of those bacteria were MDR. All examined geospatial factors were associated with MDR bacteria meat contamination. After adjustment for year and meat type, we found higher prevalence of MDR contamination among meat processed in the south (relative adjusted prevalence ratio [aPR] 1.35; 95% CI 1.06–1.73 when compared to the next-highest region), sold in Maryland (aPR 1.12; 95% CI 0.95–1.32 when compared to the next-highest state), and shipped from 194 to 469 miles (aPR 1.59; 95% CI 1.31–1.94 when compared to meats that traveled < 194 miles). However, sensitivity analyses revealed that New York sold the meat with the highest prevalence of MDR *Salmonella* contamination (4.84%). In this secondary analysis of NARMS data, both geographic location where products were sold and the shipping distance were associated with microbial contamination on retail meat.

## Introduction

Ingestion of pathogenic bacteria contaminated foods have been linked to life-threatening infections, notably from *Salmonella* and *Campylobacter* spp.^1,2^. Salmonellosis and campylobacteriosis cases cause approximately 2.36 million illnesses every year in the United States^3–6^. For severe cases which do not self-limit, antibiotics are a mainstay of clinical treatment^7,8^. However roughly 400,000 (17%) salmonellosis and campylobacteriosis infections are resistant to antibiotics^9^. Even more concerning is the development of multidrug-resistant (MDR) bacteria, defined as those resistant to more than three antibiotic classes, which limits potential treatment options for infected individuals^10^.

The animal agriculture industry has been associated with foodborne illness in humans via practices that result in contamination of animal protein food commodities^11,12^. One of the largest producers and consumers of meat in the world, the United States produced roughly 48 billion kg (110 billion pounds) of red meat and poultry in 2019^13^. This translates roughly to 97 kg (222 pounds) of meat per person annually^14^. To deliver meat to consumers, the animal agriculture industry ships livestock from producers to processors and processors to retailers, a route known as the farm to fork pathway. This series of steps might result in long distance travel, as far as 20,400 km (12,500 miles) for a single meat product^15^.

Bacterial pathogens and MDR bacteria have breached all segments of the farm-to-fork pathway. During rearing, animals harbor and are sometimes infected by bacteria^16^. Despite husbandry practices that restrict antibiotic use in animals, MDR bacteria persist in swine^17^, beef cattle^18^, and poultry^19^. After growing to a market-desired weight, animals are shipped to processor facilities, where they are harvested, packaged, and shipped again for purchase.

Processor facilities hold an intermediary role between farm and fork. During animal harvest, specific steps such as defeathering, evisceration, polishing, and scalding, might contaminate meat products with bacteria associated with the slaughtered animals^20–24^ or from the processor environment itself^25–28^. After products are packaged, retail meat are shipped up to 1800 km (1100 miles) to retail for human consumers^15^.

Contamination with pathogenic organisms is common among retail meat; as many as 37–91% of meats in the United States could harbor viable pathogenic bacteria^29–31^, while opportunistic-pathogens and indicator bacteria such as *Escherichia coli* (*E. coli*) and *Enterococcus* spp. might contaminate meat and expose consumers to antimicrobial resistance genes^32–34^. Although retail meats can ship ship long distances, few studies have investigated the relationship between distance and presence of measurable bacterial contamination nor its antimicrobial susceptibility profile. Of those that have examined this relationship, one assumed that the relationship between shipment distance and contamination was negligible based on existence of regulations that require shipping containers to maintain ideal meat storage temperatures^35^. Another observational study indicated that shifts in transport temperature could stimulate growth^36^.

Retail meat contaminated with MDR bacteria can cause significant illness and spread antimicrobial resistance genes, thus risk factors that might expose consumers and handlers of retail meat to bacterial contamination should be investigated. The purpose of this study was to describe factors related to processor origin, retailer destination, and shipment distance that could result in consumer exposure to MDR bacteria in retail meat and contribute to human illness.

## Materials and methods

### Data

#### National Antimicrobial Resistance Monitoring System

We downloaded the publicly available National Antimicrobial Resistance Monitoring System (NARMS) dataset from the U.S. Food and Drug Administration (FDA), released on November 22, 2019, which catalogued meat samples collected during 2005–2017. The FDA portion of the NARMS coalition publicizes antimicrobial susceptibility information for pathogenic and indicator bacteria isolated from retail chicken breasts, ground turkey, ground beef, and pork chops purchased at grocery stores in 19 participating states (as of 2005, although states have been joined and left over time)^37^. Metadata available in this database included antimicrobial susceptibility results by isolate, sample meat type (i.e. beef, chicken, pork, or turkey), year when meat was purchased, and state where meat was purchased. Beyond the contents of the publicly available dataset, we acquired retail meat establishment numbers (e-numbers) and retailer address of purchase via a Freedom of Information Act (FOIA) request. Only years 2012–2014 contained relevant data to analyze geospatial risk factors that overlapped with the publicly available NARMS dataset.

#### Meat, poultry, and egg product inspection

The Food Safety and Inspection Service (FSIS) of the United States Department of Agriculture (USDA) curates the Meat, Poultry, and Egg Product Inspection (MPI) Directory, a registry of e-numbers, processor company names, addresses and contact information, date of certification, and products that USDA FSIS certified-processors handle. Because the MPI compiles a list of processors currently certified, FSIS representatives were consulted to obtain the metadata information from processors that had previously received certification any time from 2010 to 2018. Individual retail meat samples with e-number data were queried in the USDA’s MPI dataset to match processor identities with physical addresses. E-numbers with matches from the cross-referenced MPI dataset had corresponding physical addresses, which we linked with the NARMS dataset. Using physical addresses, new variables were synthesized to group processors at the state and region levels. Processors were aggregated into the USDA-Agricultural Research Service (ARS) defined regions: midwest, northeast, south, and west^38^. Samples without matched physical addresses were excluded from the main analysis.

#### Geospatial information software

Although NARMS does not collect information regarding the farm location where the animal was reared, NARMS does collect information regarding processor location and final retail store before consumer purchasing. The distance that retail meats were shipped from processor to final retail was simulated using ESRI ArcGIS software under the assumption that refrigerated tractor-semitrailers deliver retail meat (ArcGIS [GIS software]. Version 10.0. Redlands, CA: Environmental Systems Research Institute, Inc., 2010). The function “Connect Origins to Destinations” was employed with the “Trucking Distance” option selected. The Connect Origins to Destination function calculates the shortest route between two locations, while the “Trucking Distance” modification adjusts routes to only include roads where trucks can legally drive^39^. Models generated trucking distance in miles, which were further divided into quartiles for interpretability.

The Connect Origins to Destinations function was performed in GIS on available e-numbers. Because 19 meat samples (0.17%) were processed outside of the continental United States (e.g., Alaska, Hawaii, Guam) the Trucking Distance models could not be available, and those meat samples were excluded from the analysis. *Post hoc*, establishment addresses were aggregated by USDA ERS regions to maintain statistical power.

### Data missingness

A missing data analysis was performed to identify each variable’s propensity to predict missingness of the models’ matched e-number categorization—coded *matched*, *unmatched*, and *not available* (NA). As a sensitivity analysis, we performed missing imputation by chained equations (see Section “Sensitivity analyses”).

### Statistical analysis

Statistical analyses were conducted to investigate MDR bacterial contamination among retail meat collected in the NARMS during years 2012–2014. Descriptive analyses were performed to enumerate meat sampled from specific states and the U.S. region from where it was processed and calculated the proportion of these meat which exhibited a MDR phenotype. We investigated if the proportion of MDR bacteria changed by year sampled, meat type, and distance the retail meat was shipped as well as by meat type and bacteria genera (among contaminated meat samples).

Unadjusted and multivariable log-binomial generalized linear regression models were developed to evaluate if geospatial features were associated with the prevalence of MDR bacterial contamination, with adjustment for meat type and year collected. For state-sampled and region-processed features, the state or region with the largest sample size were designated as the referent. Stata statistical software program was used to perform all statistical analysis (StataCorp. 2019. Stata Statistical Software: Release 16. College Station, TX: StataCorp LLC).

#### Sensitivity analyses

We performed several sensitivity analyses. First, we investigated MDR prevalence among contaminated meat as opposed to all meat regardless of contamination. This resulted in a dataset with only meat samples from which bacteria were isolated (i.e., contaminated samples); we refer to this as the “resisters.” Contaminated retail meat samples were coded as either contaminated with MDR bacteria or non-MDR bacteria, and unadjusted and multivariable models evaluated risk factors for MDR bacterial contamination among contaminated meat samples. Additional stratified models were constructed to investigate how bacteria genera and meat type might modify associations between risk factors and MDR bacterial contamination.

Retail meat collection and bacteria isolation protocols differ dependent on the NARMS state. All states tested retail meat for *Salmonella* contamination, however only Georgia, Oregon, Maryland, and Tennessee tested retail meat for all bacteria genera—*Campylobacter*, *E. coli, Enterococcus*, and *Salmonella.* Other states were only required to test poultry samples for *Campylobacter* contamination^40^. Therefore, sensitivity analyses were performed to investigate MDR bacterial contamination risk factors among retail meats in (1) the four states which cultured for all four bacteria genera, (2) *Salmonella* isolates among all meat samples, and (3) *Campylobacter* isolates among poultry samples.

To account for missing data, we performed a sensitivity analysis to compare results from the primary, complete case analysis, with those estimated with an imputed dataset. Multiple imputation by chained equations was performed using the *mice* package, version 3.15.0, in R statistical computing program (RStudio Team (2015). RStudio: Integrated Development for R. RStudio, Inc., Boston, MA URL http://www.rstudio.com/). Missing values were regressed from several variables, including bacterial contamination, bacteria MDR status, number of classes of resistance for those bacteria, date of collection, cut of meat, USDA-certified Organic label on meat, USDA-certified meat processor, raised without antibiotic label status, meat type, state collected, region processed, if meat moved across state lines, the longitude and latitude of where the meat was sampled and where the meat was processed, and the distance that the meat traveled between processor and retail store in miles. Five iterations of the multiple imputation were analyzed with pooled results.

### Processor to retail maps

A series of maps were developed to depict the shipment of individual retail meat types within the NARMS states during 2012–2014, using the *maps* package, version 3.4.1, in RStudio. Using locations gathered through MPI queries and the NARMS database, we aggregated processors and retailers to the state level. Individual retail meat collections were stratified based upon their path, from processor to retail, and connected with an arched line. Those pathways were then evaluated for prevalence of MDR bacterial contamination*.*

## Results

### Processor identity and retail meat shipment

 During 2012–2014, NARMS collected 18,512 individual retail meat samples that contained information on processor and retail store identity. Missing information for processor data reduced the meat samples that were analyzed. Many e-numbers could not be matched to a distinct processer (n = 7255; 39%), and most of the unmatched processors had some iteration of “not available” listed under the e-number feature (n = 6767; 93%). The other 488 (3%) meat samples were processed at facilities which could not be matched to a known MPI establishment code. A small percentage of e-numbers could be uniquely identified with the insertion of a single missing digit, and were assigned to corresponding processors in MPI (n = 370; 2%). Few meat samples were excluded from analyses because their processor was in a state or territory where shipment solely by truck was infeasible (i.e., Alaska, n = 1; Guam, n = 2; and Hawaii, n = 12). Final included meat samples totaled 11,243. Investigating how data were missing, we discovered that meat type and Organic status consistently accounted for missingness. (Supplementary Table 1). We also found that the year in which the meat sample was purchased and the MDR outcome might have accounted for missingness in the NARMS dataset.

**Figure 1:**
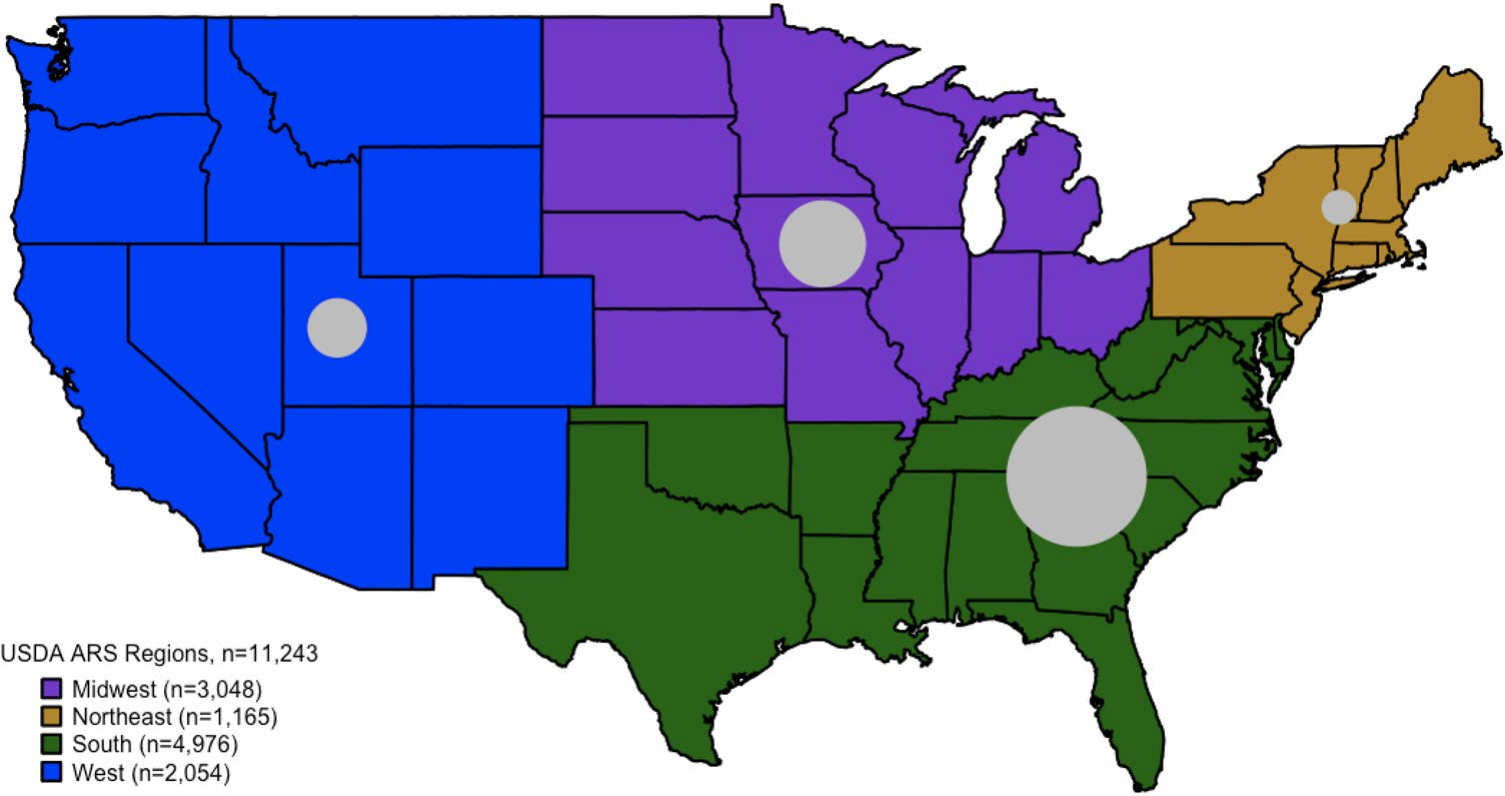
Processor locations were aggregated by USDA ARS regions. Size of grey circles reflects the number of NARMS collected retail meat processed in a specific region. Processors located in the south region handled (44.3%) of all meat collected by NARMS from 2012 to 2014. Source: United States Food and Drug Administration National Antimicrobial Resistant Monitoring System.

Among the 11,243 samples, the number of retail meats with traceable e-numbers increased each year from 2012 to 2014: 3127 samples in 2012, 3991 samples in 2013, and 4139 samples in 2014. Ground turkey were collected most frequently, with 4152 (36.9%) samples, followed by chicken breast at 3736 (33.2%), ground beef at 2164 (19.3%) and pork chops at 1191 (10.6%) (Table 1). Overall, the 14 NARMS states had meat samples with identifiable processor codes from 537 (Connecticut) to 1083 (Georgia) retail meat samples. The region where most meat samples were processed was in the south (44.3% of samples), followed by the midwest (27.0%), the West (18.3%), and the Northeast (10.4%) (Fig. 1). The average distance that meat traveled from processor to retailer was 619.25 miles (~ 997 km) (Table 1).

**Table 1:** Log-binomial analysis of bacteria-contaminated retail meat and risk factors collected by NARMS, 2012–2014.

Variables	Meat samples, n	Overall bacterial contamination, n (%)		Unadjusted prevalence of contamination, PR (95% CI)	Adjusted prevalence of contamination (year and meat type), aPR (95% CI)
Year					
2012	3125	1582	(50.62)	REF^†^	REF
2013	3987	1642	(41.18)	0.81 (0.77–0.86)**	–
2014	4131	1567	(37.93)	0.75 (0.71–0.79)***	–
Meat type					
Chicken breast	3736	2212	(59.21)	REF	REF
Ground turkey	4152	1481	(35.67)	0.60 (0.57–0.63)***	–
Ground beef	2164	674	(31.15)	0.52 (0.49–0.56)***	–
Pork chop	1191	424	(35.60)	0.60 (0.55–0.65)***	–
State sampled					
Georgia	1083	1078	(99.54)	REF	REF
Oregon	953	869	(91.19)	NE	NE
Minnesota	906	121	(13.36)	NE	NE
New York	889	155	(17.44)	NE	NE
Pennsylvania	868	150	(17.28)	NE	NE
Colorado	852	130	(15.26)	NE	NE
Tennessee	833	798	(95.80)	NE	NE
Maryland	810	790	(97.53)	NE	NE
Washington	776	103	(13.27)	NE	NE
New Mexico	735	144	(19.59)	NE	NE
California	721	136	(18.86)	NE	NE
Missouri	670	121	(18.06)	NE	NE
Louisiana	610	116	(19.02)	NE	NE
Connecticut	537	83	(15.46)	NE	NE
Region processed					
South	4976	2868	(57.64)	REF	REF
Midwest	3048	876	(28.74)	0.53 (0.50–0.57)***	0.63 (0.59–0.67)***
West	2054	765	(37.24)	0.69 (0.65–0.73)***	0.76 (0.71–0.81)***
Northeast	1165	464	(39.83)	0.74 (0.68–0.80)***	0.88 (0.81–0.95)***
Distance traveled quartiles, (mi)					
0–193	2867	1230	(42.90)	REF^†^	REF^†^
194–468	2604	1427	(54.80)	1.28 (1.21–1.35)***	1.14 (0.95–1.38)
469–909	2769	1136	(41.03)	0.96 (0.90–1.02)	1.16 (0.99–1.35)
910–2948	3003	999	(33.27)	0.72 (0.72–0.83)***	0.95 (0.82–1.10)

Prevalence of overall bacteria and MDR bacteria contaminated retail meat was calculated by dividing the number of total or MDR bacteria contaminated samples over the total meat samples. Unadjusted models were evaluated to determine prevalence of MDR bacteria among all meat samples (human exposure) and MDR bacteria among contaminated meat samples (resister). Source: United States Food and Drug Administration National Antimicrobial Resistance Monitoring System.

*REF* referent group, *PR* Prevalence Ratio, *CI* 95% Confidence Intervals, *aPR *Adjusted Prevalence Ratio, *MDR* multidrug resistant, *NE* not estimable.

*P < 0.05, **P < 0.01, ***P < 0.001, ^†^*P*-trend < 0.05.

### Retail meat contamination analysis

Approximately 42.6% of collected meat demonstrated detectable bacteria, with 21.1% of cultured samples (7.4% of total isolates) being MDR. Overall bacterial contamination decreased from 50.6% in 2012 to 37.9% in 2014, and MDR bacterial contamination rates declined over the same interim (19.0% in 2012, 18.2% in 2013, and 15.0% in 2014). Poultry (chicken and turkey) retail meats had the highest bacterial meat contamination prevalence, with both overall bacteria and MDR bacteria. Among poultry, chicken breast samples had the highest overall bacterial contamination (59.4%), while ground turkey had the highest MDR prevalence among contaminated product (38.1%). Ground beef had the lowest overall bacterial contamination (31.7%) and MDR bacteria (4.0%) among contaminated meat (Table 1).

Retail meats had varied prevalence of bacterial and MDR contamination by state. Meats collected in Washington and Minnesota had among the lowest prevalence of overall bacterial contamination levels (13.3% and 13.4%, respectively), while California-purchased retail meat samples had the lowest prevalence of contaminated meats with MDR bacteria at 3.0%. Retail meat in Maryland had among the highest contaminant levels both for overall bacteria (97.4%) and MDR bacteria (30.0%). Regionality where contaminated meat samples were purchased also varied. The lowest proportion of overall contaminated meat and MDR contaminated meat was purchased from the midwest (15.1% and 6.3%). However, the highest proportion of overall bacterial contamination and MDR bacteria among contaminated meats were not purchased from the same region: meat purchased from the south had the highest proportion of overall bacteria contaminated meat (83.35%), while the highest contaminated meat with MDR bacteria was in the northeast (23.2%).

Unadjusted and adjusted regression analyses revealed that all variables—meat type, year, state purchased, region processed, distance between retail meat processor and retailer—were significantly associated with MDR bacterial contamination among collected meat samples (Table 2). Ground turkey had a significantly higher prevalence of MDR bacterial contamination than chicken breast samples (PR 2.25, 95% CI 1.94–2.6). Among all meat samples, annual prevalence of MDR bacterial contamination decreased by 22% from 2012 to 2013 (PR 0.78, 95% CI 0.67–0.91) and 41% from 2012 to 2014 (PR 0.59, 95% CI 0.50–0.70) (Table 2).

**Table 2:** Log-binomial analysis of MDR bacteria-contaminated retail meat and risk factors, years 2012–2014.

Variables	Meat samples, n	Overall bacterial contamination, n (%)		MDR, n (%)		Unadjusted primary outcome: consumer exposure to MDR bacteria, PR (95% CI)	Adjusted primary outcome, aPR (95% CI)	Secondary outcome: resistome, PR (95% CI)	Adjusted secondary outcome, aPR (95% CI)
Year									
2012	3125	1582	(50.62)	301	(9.63)	REF^†^	–	REF^†^	–
2013	3987	1642	(41.18)	299	(7.50)	0.78 (0.67–0.91)**	–	0.96 (0.83–1.11)	–
2014	4131	1567	(37.93)	235	(5.69)	0.59 (0.50–0.70)***	–	0.79 (0.67–0.92)**	–
Meat type									
Chicken breast	3736	2212	(59.21)	225	(6.02)	REF	–	REF	–
Ground turkey	4152	1481	(35.67)	562	(13.54)	2.25 (1.94–2.61)***	–	3.73 (3.24–4.29)***	–
Ground beef	2164	674	(31.15)	27	(1.25)	0.21 (0.14–0.31)***	–	0.39 (0.27–0.58)***	–
Pork chop	1191	424	(35.60)	21	(1.76)	0.29 (0.19–0.45)***	–	0.11 (0.32–0.75)***	–
State sampled									
Georgia	1083	1078	(99.54)	196	(18.10)	REF	REF	REF	REF
Oregon	953	869	(91.19)	179	(18.78)	1.04 (0.86–1.25)	0.93 (0.79–1.10)	1.13 (0.94–1.36)	NE
Minnesota	906	121	(13.36)	11	(1.21)	0.07 (0.04–0.12)***	0.06 (0.2–0.12)***	0.51 (0.29–0.91)*	NE
New York	889	155	(17.44)	45	(5.06)	0.28 (0.20–0.38)***	0.24 (0.18–0.33)***	1.60 (1.21–2.11)**	NE
Pennsylvania	868	150	(17.28)	27	(3.11)	0.17 (0.12–0.25)***	0.15 (0.10–0.22)***	0.99 (0.69–1.43)	NE
Colorado	852	130	(15.26)	7	(0.82)	0.05 (0.012–0.10)***	0.05 (0.02–0.10)***	0.30 (0.14–0.62)***	NE
Tennessee	833	798	(95.80)	139	(16.69)	0.92 (0.76–1.12)	0.83 (0.69–1.00)	0.96 (0.79–1.17)	NE
Maryland	810	790	(97.53)	182	(22.47)	1.24 (1.04–1.48)*	1.12 (0.95–1.32)	1.27 (1.06–1.52)*	NE
Washington	776	103	(13.27)	5	(0.64)	0.04 (0.01–0.09)***	0.03 (0.01–0.08)***	0.27 (0.11–0.63)**	NE
New Mexico	735	144	(19.59)	12	(1.63)	0.09 (0.05–0.16)***	0.08 (0.05–0.15)***	0.46 (0.26–0.80)**	NE
California	721	136	(18.86)	4	(0.55)	0.03 (0.01–0.08)***	0.02 (0.01–0.07)***	0.16 (0.06–0.43)***	NE
Missouri	670	121	(18.06)	4	(0.60)	0.03 (0.01–0.09)***	0.03 (0.01–0.08)***	0.18 (0.07–0.48)**	NE
Louisiana	610	116	(19.02)	6	(0.98)	0.05 (0.02–0.12)***	0.05 (0.02–0.12)***	0.28 (0.13–0.63)**	NE
Connecticut	537	83	(15.46)	18	(3.35)	0.19 (0.12–0.30)***	0.05 (0.02–0.10)***	0.30 (0.14–0.62)**	NE
Region processed									
South	4976	2868	(57.64)	457	(9.18)	REF	REF	REF	REF
Midwest	3048	876	(28.74)	241	(7.91)	0.86 (0.74–1.00)*	0.68 (0.58–0.79)***	1.62 (1.41–1.85)***	0.82 (0.71–0.93)**
West	2054	765	(37.24)	74	(3.60)	0.39 (0.31–0.50)***	0.74 (0.58–0.94)*	0.80 (0.63–1.01)	0.72 (0.58–0.90)**
Northeast	1165	464	(39.83)	63	(5.41)	0.59 (0.46–0.76)***	0.67 (0.52–0.86)**	0.57 (0.45–0.72)***	0.56 (0.45–0.71)***
Distance traveled quartiles, (mi)									
0–193	2867	1230	(42.90)	144	(5.02)	REF^†^	REF^†^	REF^†^	REF^†^
194–468	2604	1427	(54.80)	224	(8.60)	1.71 (1.40–2.10)***	1.59 (1.31–1.94)***	1.31 (1.08–1.59)**	1.01 (0.85–1.21)
469–909	2769	1136	(41.03)	235	(8.49)	1.69 (1.38–2.07)***	1.09 (1.89–1.34)	1.76 (1.46–1.13)***	0.83 (0.69–1.00)*
910–2948	3003	999	(33.27)	232	(7.73)	1.54 (1.26–1.88)***	0.85 (169.-1.03)	1.96 (1.62–2.38)***	0.88 (0.73–1.05)

Prevalence of overall bacteria and MDR bacteria contaminated retail meat was calculated by dividing the number of total or MDR bacteria contaminated samples over the total meat samples. Unadjusted models were evaluated to determine prevalence of MDR bacteria among all meat samples (human exposure) and MDR bacteria among contaminated meat samples (resister). Source: United States Food and Drug Administration National Antimicrobial Resistance Monitoring System.

*REF* referent group, *PR* Prevalence Ratio, *CI* Confidence Intervals, *aPR *Adjusted Prevalence Ratio.

*P < 0.05, **P < 0.01, ***P < 0.001, ^†^*P*-trend < 0.05.

In comparison to meat samples collected in Georgia, retail meat from Maryland had the highest prevalence of both MDR bacteria contamination among all meat samples (PR 1.27, 95% CI 1.06–1.52) and prevalence of MDR bacteria contamination among contaminated meat (PR 1.24, 95% CI 1.04–1.48). California had the lowest contamination in both comparisons (PR 0.03, 95% CI 0.01–0.08 and PR 0.16 95% CI 0.06–0.43, respectively) (Table 2). Findings were consistent in multivariable analyses after adjusting for meat type and year collected. The four states that sample indicator bacteria had the highest proportion of MDR bacterial contamination: Maryland (PR 1.24, 95% CI 1.04–1.48), Oregon (PR 1.04, 95% CI 0.86–1.25), Georgia (referent), and Tennessee (PR 0.92, 95% CI 0.76–1.12). The next largest prevalence of MDR bacteria in retail meat was in New York (PR 0.28, 95% CI 0.20–0.38). However, the resister outcome indicated that despite the absence of indicator bacteria, the states with highest prevalence for MDR bacteria among contaminated meats were New York (PR 1.60, 95% CI 1.21–2.11), which had the highest prevalence, and Maryland (PR 1.27, 95% CI 1.06–1.52), the second highest (Table 2). The corresponding imputed models did not diverge substantially from these findings (Supplementary Table 2).

Because NARMS states adopt protocols which isolate different bacteria, sensitivity analyses were performed to investigate *Salmonella* isolates among all states in all meat, and *Campylobacter* among all states in poultry meats. Findings were only slightly changed where the four states which tested for all four bacterial genera had among the highest MDR *Salmonella* contaminated meats within both outcomes*.* However, because of the low MDR *Campylobacter* prevalence, only Maryland, New York, and Pennsylvania prevalence were estimable, although none were significantly different from the Georgia referent.

### Meat processor analysis

Overall bacterial contamination and MDR bacterial contamination where meats were regionally processed varied less, from 22.5% and 3.6% in the midwest, to 57.6% and 9.2% in the south (Table 1). NARMS collected the most amount of retail meats processed in the south (44.3%) (Table 1). As the referent group, meat processed in the south had the highest prevalence of overall bacteria (57.6%) and MDR bacterial contamination (9.18%) (Table 1). Processors located in Delaware and North Carolina had the highest proportion of meat samples with MDR bacterial contamination at 21.4% (n = 78/365) and 14.0% (n = 130/927), respectively. Alternatively, consumers who purchase retail meat from the northeast were least likely to be exposed to meat contaminated with MDR bacteria (PR 0.39, 95% CI 0.31–0.50). Findings were consistent in multivariable analyses after adjustment of 1) meat type and 2) meat type and year collected. However, in terms of the resister outcome, the midwest had the highest prevalence of MDR bacterial contamination (PR 1.62, 95% CI 1.41–1.85) (Table 2). However, after adjustment for meat type and year, meat processed in the south were noted to have the highest prevalence for MDR bacterial contamination in the resister outcome. Sensitivity analyses that evaluated MDR *Salmonella* contamination found that meats processed in the south had the highest prevalence of contamination in both outcomes (Supplementary Table 3).

### Distance shipped influences factors of MDR

**Figure 2:**
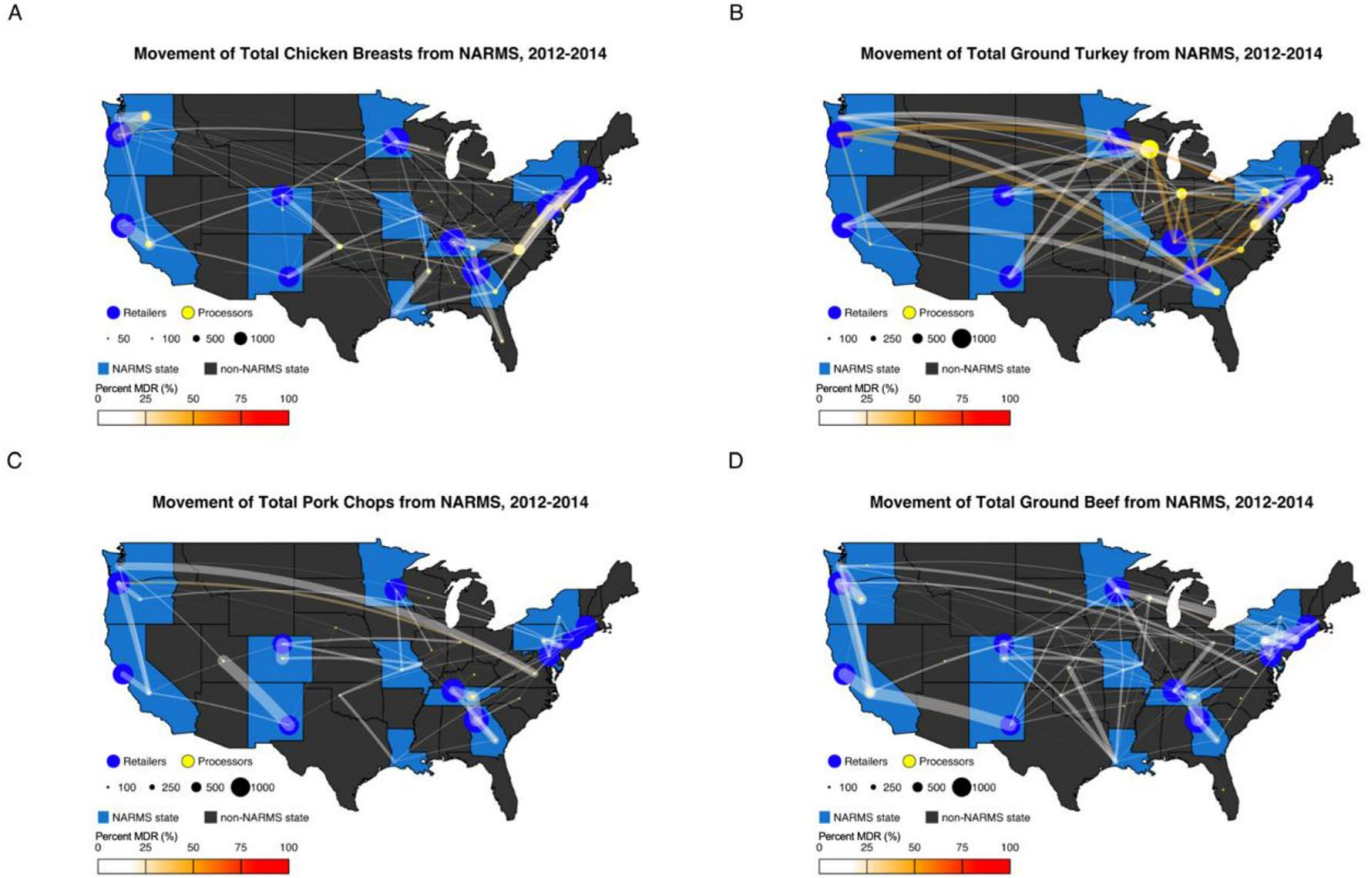
Retail Meat travels across the United States, from where they are processed (yellow circle) to where they are ultimately purchased (blue circles). Processors were binned by state and located by state centroid for easier visualization. Retailers were similarly binned by state and located approximate to state health department locations where samples were analyzed. NARMS states are filled in blue, whereas non-NARMS states are grey. The prevalence of MDR bacteria from origin to destination (aggregated by state) was also depicted by the hue of the arc, white reflecting 0% contamination and red indicating 100% contamination. Chicken breasts (**A**) traveled the shortest distances with the least MDR bacterial contamination. However, ground turkey (**B**) traveled the furthest and had the highest prevalence of MDR bacterial contamination. Ground beef products (**D**) were processed from the most states (n = 31) and had the lowest prevalence of MDR bacterial contamination. Pork chops (**C**) were processed in 23 states with the highest MDR bacterial contamination along the Oregon-Virginia pathway. Source: United States Food and Drug Administration National Antimicrobial Resistance Monitoring System.

The 11,234 meat samples with matched processor location and retailer location are depicted in Fig. 2, aggregated by state. These maps illustrate that whole cut products (pork and chicken) had a lower MDR bacterial contamination prevalence compared to the ground products (beef and turkey) on any individual route. Among all origin to destination movements by state, the highest prevalence of MDR bacterial contamination occurred within pork chops. Among all meat types, the highest prevalence of MDR bacterial contamination were ground turkey products which traveled from Virginia to Maryland (73.7%), Virginia to Georgia (57.1%), Wisconsin to Maryland (57.1%), and Virginia to Tennessee (54.8%).

Shipping distance was dependent upon region. Midwest processors shipped 46% of their meats within the fourth distance quartile, Northeast processors shipped 37% and 36% of their meats in the first and second distance quartile respectively, Southern processors shipped 34% and 33% of their meats in the second and third distance quartiles, and Western processors shipped 49% of their meats within the first distance quartile (Supplementary Table 4). The prevalence of MDR bacterial contamination on meat increased about 60% for samples transported > 194 miles (PR 1.63, 95% CI 1.33–1.99), but the prevalence remained statistically similar for higher quartiles, although the trend was statistically significant (*P* < 0.001) (Table 1). Multivariable analysis adjusting for year collected and meat type corroborated the results from the unadjusted analyses (Table 1, trend *P* < 0.001). However, resister outcome analysis found increasing prevalence of MDR bacterial contamination.

Processor regions that shipped retail meat the furthest was the midwest region (median = 839.5 miles). Conversely, processors located in the West traveled the shortest distances (median = 200 miles). The processors in the south—associated with the highest prevalence of MDR bacterial contamination (9.2%) on retail meat—transported 33% of retail meat, which traveled a median of 464.9 miles (Supplementary Table 5).

### Stratified analyses

Descriptive analyses by bacterial genus (Supplementary Table 6) and meat type (Supplementary Table 7) were performed to explore the potential for these variables to modify the relationship between geospatial risk factors and MDR bacterial contamination. Prevalence of MDR bacterial contamination decreased among all meat types except pork chops, which increased slightly from 3.6 to 4.5%. Southern processors had the highest MDR bacterial contamination among chicken breast (11.1%), ground turkey (45.3%), and ground pork (6.0%) (Supplementary Table 7). The states with largest MDR bacteria contaminated meat differed by bacteria genus. New York had the highest MDR *Salmonella* prevalence (48.0%), Pennsylvania had the highest MDR *Campylobacter* contamination levels (3.4%), Oregon had the highest MDR *E. coli* contamination (38.9%), and Maryland had the highest MDR *Enterococcus* contamination (3.0%). Only Southern processers had MDR *Campylobacter* contaminated meat (2.1%). *Campylobacter* had among the lowest proportion of MDR bacterial contamination among all meat sampled, regardless of risk factor. Interestingly, the proportion of MDR bacterial contamination was similar across distances for both *Salmonella* and *Campylobacter* contamination (Supplementary Table 6).

## Discussion

When consumers handle, prepare, or eat retail meat, they risk bacterial exposure. Consumer exposures of pathogenic bacteria like *Salmonella *and *Campylobacter* could lead to foodborne illness. Although some studies have described the epidemiology of bacterial contamination on retail meat, no studies to our knowledge have used a national database to explore geospatial risk factors of MDR bacterial contamination on retail meat. This study has detailed sources of retail meat sold locally and transcontinental, which could alter the MDR bacterial contamination. Retail meats included in this analysis were neither processed nor distributed equally across the United States, and we identified a potential association between distance traveled and prevalence of MDR bacteria, which might suggest a role for shipment distance in MDR bacterial contamination.

This study corroborated findings previously reported in the NARMS reports: overall bacterial and MDR bacterial contamination of meat decreased from 2012 to 2014 (50.6% to 37.9% and 9.6% to 8.6%, respectively). This represents a MDR bacteria reduction of 22% from 2012 to 2013 and 41% from 2012 to 2014 (Table 1). Several mechanisms might have resulted in reductions such as might improvements in husbandry practices, such as vaccination, or processor protocols that limit bacterial contamination. Likewise, decreases might represent the industry’s anticipation of federal policies to reduce antimicrobial resistant and MDR bacteria prevalence in the U.S. food system^41,42^.

Across analyses, findings demonstrated strong associations with meat type and MDR bacterial contamination prevalence. Poultry (i.e., turkey and chicken) demonstrated significantly higher prevalence of MDR bacterial contamination, with ground turkey having the highest prevalence—2.7-fold higher than chicken breast. Among ground products, ground turkey products had an 8.5-fold greater prevalence of MDR bacterial contamination than ground beef. The additional grinding steps that incorporate hundreds of animals from different countries into a single batch of ground meat might contribute to an a larger prevalence of MDR bacterial contamination^43–47^.

Limited evidence exists to explain regional differences associated with MDR bacterial contamination on retail meat. Upstream factors, such as husbandry practices are shown to vary the bacteria harbored on the animals that enter the processor facilities, which could alter bacterial contamination on the final product^34^. For example, some states have many more USDA-certified Organic farms than others^48^. States also have some state-specific humane slaughter laws^49^ and inspection programs^50^. Although microbial contamination of retail meat is known to occur from slaughtering procedures, any differences in contamination as a result of state-specific humane laws and inspection programs is unknown and therefore an opportunity for further research. Regardless of the underlying reason, e-numbers, when available and transparent, could provide traceback information to identify individual processor entities and their facility locations and potentially be used to intervene and decrease exposures from MDR bacteria contaminated retail meat.

From production to retail, meat products are transported lengthy distances^15^. NARMS meat samples corroborate this: retail meat traveled on average 470 miles solely between processing plants and retailers. Increased distance was associated with increased MDR bacterial contamination both among all retail meat samples and those contaminated (Table 1; *P* < 0.05). Multivariable analyses that explored associations of risk factors (1) shipment distances and (2) processor region with MDR bacterial contamination stayed consistent, which could suggest that both shipment distances and region processed are important risk factors independently. One biologically plausible hypothesis is that shipment conditions might promote bacterial growth on contaminated retail meats resulting from temperature inconsistencies among refrigerated trucks. Mandated by the USDA, refrigerated trucks must be at 35–40°F or lower—temperatures at which bacteria cannot replicate^39,51,52^. However, longer distances and subsequently longer transit time that refrigerated trucks travel increase the concern for gaps in a continuous cold chain and humidity control^53^. For example, temperature and humidity variance along transit could challenge bacteriostatic conditions^54^. After transportation, retailer conditions might similarly impact bacterial growth on retail meat products. One study found that the temperature control where retailers’ store their retail meat might contribute to bacterial growth^55^. To investigate causality for these hypotheses, more data are imperative to investigate realities of temperature variation in the farm to fork pathway. Temperature and humidity measurements in refrigerated trucks and retailer refrigerators could provide insight into these relationships. However, additional experiments should be conducted to further investigate these associations, as sensitivity analyses contradicted results.

Analyses performed are only as strong as the data used, albeit NARMS is the most comprehensive database of retail meat isolates collected in the United States. Because of the limitations detailed below, we encourage that hypotheses are generated from our findings to confirm or deny statistically significant associations.

Several factors limit our findings’ generalizability. First, only four states measured *Salmonella*, *Campylobacter*, *E. coli*, and *Enterococcus* spp., and regardless, *Campylobacter* was only assessed in poultry products. Therefore, *Salmonella*, and to a lesser extent, *Campylobacter* might be oversampled in the NARMS dataset. However, sensitivity analysis to restrict states that cultured all bacteria genus demonstrated had similar findings to analysis of the combined dataset. Similarly, analyses findings from the *Campylobacter-*specific analyses might be difficult to generalize based on a low MDR prevalence overall (about 1%). Further, because NARMS is a national database with only 14 unique states represented, findings should not be generalized to the entire United States. The FDA specifically notes this point in addition to highlighting the small sampling size, both of which introduce bias that could hamper the interpretability of results^56^.

Secondly, significant missing data (39%) likely bias our estimations and interpretations in our complete case analysis. Although results did not significantly change when accounting for missingness via multiple imputation by chained equations, e-numbers listed as NA might have specific reasons for missingness other than data entry error (i.e., *missing not at random*), thus available explanatory variables might not completely account for missingness^57^. Unavailability of e-numbers might have been due to repackaging or subsequent meat processing (e.g., cutting and grinding) at the retail level. Retailers are required to maintain records of e-numbers but are not required to label meat packages with e-numbers due to an exemption status in accordance with 9 CFR 317.2(i) and 381.123. Therefore, retail meat collected in the NARMS system might not have had e-numbers to report. Otherwise, compromised meat packages or human error might be a source of missing data, since data entry is performed by staff the state level. Consequently, the sole solution to overcome this challenge requires NARMS to improve its standards and acceptable missingness during retail meat collection and data acquisition.

Lastly, the available data are now dated. Timing was such that it was not otherwise possible to obtain updated data and relink the disparate datasets. We weighed the time intensity and resources to continue this work and conduct additional analysis. We encourage researchers to conduct future studies that investigate subsequent years’ data and endeavor through time-consuming legal processes (i.e., FOIA requests) to obtain potentially sensitive, publicly unavailable data.

## Conclusions

In this secondary analysis of data obtained by a government-led retail meat sampling monitoring system, the geographic location where consumers buy retail meat, where meat was processed, and the distance at which the product was shipped were associated with MDR bacterial contamination of retail meat products. To ameliorate exposures to MDR bacteria from retail meat, further investigation about retail meat processing and methods to reduce bacterial contamination, particularly in the turkey industry and processors located in the south.

### Supplementary Information

Supplementary Tables.

## Data Availability

All datasets used in this analysis was publicly available from the United States government. FDA’s NARMS data can be found on their website at https://www.fda.gov/media/93325/download?attachment; USDA’s Organic Integrity database can be found on their website at https://data.nal.usda.gov/dataset/organic-integrity-database; and USDA’s Meat, Poultry and Egg Product Inspection Directory can be found on their website at https://www.fsis.usda.gov/inspection/establishments/meat-poultry-and-egg-product-inspection-directory. All datasets were accessible on September 21, 2023.

## References

[1] Chousalkar KK, Willson N-L (2022). Nontyphoidal Salmonella infections acquired from poultry. Curr. Opin. Infect. Dis..

[2] Silva J (2011). Campylobacter spp. as a foodborne pathogen: A review. Front. Microbiol..

[3] Scallan E (2011). Foodborne illness acquired in the United States-Major pathogens. Emerg. Infect. Dis..

[4] Painter JA (2013). Attribution of foodborne illnesses, hospitalizations, and deaths to food commodities by using outbreak data, United States, 1998–2008. Emerg. Infect. Dis..

[5] Beshearse E (2021). Attribution of illnesses transmitted by food and water to comprehensive transmission pathways using structured expert judgment, Unites States. Emerg. Infect. Dis..

[6] Collier SA (2021). Estimate of burden and direct healthcare cost of infectious waterborne disease in the United States. Emerg. Infect. Dis..

[7] Crum-Cianflone NF (2008). Salmonellosis and the gastrointestinal tract: More than just peanut butter. Curr. Gastroenterol. Rep..

[8] Yang Y (2019). A historical review on antibiotic resistance of foodborne campylobacter. Front. Microbiol..

[9] Solomon, S. L., & Oliver, K. B. *Antibiotic Resistance Threats in the United States, 2019*. (2019). 10.15620/cdc:82532

[10] Magiorakos A-P (2012). Multidrug-resistant, extensively drug-resistant and pandrug-resistant bacteria: An international expert proposal for interim standard definitions for acquired resistance. Clin. Microbiol. Infect..

[11] Innes GK (2020). External societal costs of antimicrobial resistance in humans attributable to antimicrobial use in livestock. Annu. Rev. Public Health.

[12] Heredia N, García S (2018). Animals as sources of food-borne pathogens: A review. Anim. Nutr..

[13] Haley, M. *USDA ERS: Livestock, Dairy, and Poultry Outlook: December 2019. ERS Livestock, Dairy, Poult. Outlook* (2019).

[14] National Chicken Council. *Per Capita Consumption of Poultry and Livestock, 1960 to Forecast 2020, in Pounds*. (2021).

[15] Weber CL, Matthews HS (2008). Food-miles and the relative climate impacts of food choices in the United States. Environ. Sci. Technol..

[16] Lekshmi M, Ammini P, Kumar S, Varela MF (2017). The food production environment and the development of antimicrobial resistance in human pathogens of animal origin. Microorganisms.

[17] Österberg J (2016). Antibiotic resistance in *Escherichia coli* from pigs in organic and conventional farming in four european countries. PLoS ONE.

[18] Agga GE (2019). Persistence of antibiotic resistance genes in beef cattle backgrounding environment over two years after cessation of operation. PLoS ONE.

[19] Nhung NT, Chansiripornchai N, Carrique-Mas JJ (2017). Antimicrobial resistance in bacterial poultry pathogens: A review. Front. Vet. Sci..

[20] Rouger A, Tresse O, Zagorec M (2017). Bacterial contaminants of poultry meat: Sources, species, and dynamics. Microorganisms.

[21] Pacholewicz E, Swart A, Wagenaar JA, Lipman LJA, Havelaar AH (2016). Explanatory variables associated with campylobacter and *Escherichia coli* concentrations on broiler chicken carcasses during processing in two slaughterhouses. J. Food Prot..

[22] Wheatley P, Giotis ES, McKevitt AI (2014). Effects of slaughtering operations on carcass contamination in an Irish pork production plant. Ir. Vet. J..

[23] Saide-Albornoz JJ, LynnKnipe C, Murano EA, Beran GW (1995). Contamination of pork carcasses during slaughter, fabrication, and chilled storage. J. Food Prot..

[24] Tadesse DA (2011). Prevalence and antimicrobial resistance profile of *Campylobacter* spp. isolated from conventional and antimicrobial-free swine production systems from different U.S. regions. Foodborne Pathog. Dis..

[25] Vihavainen E (2007). Role of broiler carcasses and processing plant air in contamination of modified-atmosphere-packaged broiler products with psychrotrophic lactic acid bacteria. Appl. Environ. Microbiol..

[26] Hultman J, Rahkila R, Ali J, Rousu J, Björkroth KJ (2015). Meat processing plant microbiome and contamination patterns of cold-tolerant bacteria causing food safety and spoilage risks in the manufacture of vacuum-packaged cooked sausages. Appl. Environ. Microbiol..

[27] Wang R (2019). Biofilms and meat safety: A mini-review. J. Food Protect..

[28] Giaouris E (2014). Attachment and biofilm formation by foodborne bacteria in meat processing environments: Causes, implications, role of bacterial interactions and control by alternative novel methods. Meat Sci..

[29] Davis GS (2018). Antibiotic-resistant *Escherichia coli* from retail poultry meat with different antibiotic use claims. BMC Microbiol..

[30] Johnson JR, Kuskowski MA, Smith K, O’Bryan TT, Tatini S (2005). Antimicrobial-resistant and extraintestinal pathogenic *Escherichia coli* in retail foods. J. Infect. Dis..

[31] Miranda JM (2007). Antimicrobial resistance in *Enterococcus* spp. strains isolated from organic chicken, conventional chicken, and turkey meat: A comparative survey. J. Food Prot..

[32] Colavecchio A, Cadieux B, Lo A, Goodridge LD (2017). Bacteriophages contribute to the spread of antibiotic resistance genes among foodborne pathogens of the Enterobacteriaceae family: A review. Front. Microbiol..

[33] Zansky S (2002). From the centers for disease control and prevention. Outbreak of multi-drug resistant Salmonella Newport-United States, January–April 2002. JAMA.

[34] Innes GK (2021). Contamination of retail meat samples with multidrug-resistant organisms in relation to organic and conventional production and processing: A cross-sectional analysis of data from the United States National Antimicrobial Resistance Monitoring System, 2012. Environ. Health Perspect..

[35] Tesson V (2020). A systematic review of beef meat quantitative microbial risk assessment models. Int. J. Environ. Res. Public Health.

[36] Love DC (2020). Performance of cold chains and modeled growth of Vibrio parahaemolyticus for farmed oysters distributed in the United States and internationally. Int. J. Food Microbiol..

[37] Food and Drug Administration. *Integrated Reports/Summaries|FDA*. (2020).

[38] United States Department of Agriculture (USDA). *Regions: USDA ARS*. (2020).

[39] FSIS. *FSIS Safety and Security Guidelines for the Transportation and Distribution of Meat, Poultry, and Egg Products*. (2003).

[40] FDA, N. *The National Antimicrobial Resistance Monitoring System: Enteric Bacteria Methods*, 1–7 (2009).

[41] Food and Drug Administration (FDA). *Guidance for Industry New Animal Drugs and New Animal Drug Combination Products Administered in or on Medicated Feed or Drinking Water of Food-Producing Animals: Recommendations for Drug Sponsors for Voluntarily Aligning Product Use Conditions with GFI #2*.

[42] Center for Veterinary Medicine, Food and Drug Administration (FDA). *List of Medically Important Antimicrobial Drugs Affected by GFI #213*. https://www.fda.gov/animal-veterinary/judicious-use-antimicrobials/list-medically-important-antimicrobial-drugs-affected-gfi-213. Accessed 7 May 2019.

[43] Hu XS (2012). Estimating animal abundance in ground beef batches assayed with molecular markers. PLoS ONE.

[44] White DG, Zhao S, Simjee S, Wagner DD, McDermott PF (2002). Antimicrobial resistance of foodborne pathogens. Microb. Infect..

[45] White DG (2001). The isolation of antibiotic-resistant salmonella from retail ground meats. N. Engl. J. Med..

[46] Han F, Lestari SI, Pu S, Ge B (2009). Prevalence and antimicrobial resistance among *Campylobacter* spp. in Louisiana retail chickens after the enrofloxacin ban. Foodborne Pathog. Dis..

[47] Vikram A (2018). Similar levels of antimicrobial resistance in US food service ground beef products with and without a ‘“raised without antibiotics”’ claim. J. Food Prot..

[48] National Agricultural Statistics Service*. 2021 Certified Organic Survey*. (2022).

[49] Wisch, R. F. *Table of State Humane Slaughter Laws|Animal Legal & Historical Center*. (2006).

[50] Federal-State Audit Staff. *FSIS Audit of State Meat and Poultry Inspection Programs Fiscal Year 2022 Summary Report*. (2023).

[51] USDA. *How Temperatures Affect Food*. (2011). 10.17660/ActaHortic.2018.1192.5.

[52] Ashby, B. H. *Protecting Perishable Fods During Transport by Truck.* (US Department of Agriculture, 1995).

[53] Ruiz-Garcia L, Barreiro P, Rodriguez-Bermejo J, Robla JI (2007). Review: Monitoring the intermodal, refrigerated transport of fruit using sensor networks. Span. J. Agric. Res..

[54] Giannuzzi L, Pinotti A, Zaritzky N (1998). Mathematical modelling of microbial growth in packaged refrigerated beef stored at different temperatures. Int. J. Food Microbiol..

[55] Brown LG (2018). Food safety practices linked with proper refrigerator temperatures in retail delis. Foodborne Pathog. Dis..

[56] Food and Drug Administration (2017). Science Board Review of the National Antimicrobial Resistance Monitoring System.

[57] Madley-Dowd P, Hughes R, Tilling K, Heron J (2019). The proportion of missing data should not be used to guide decisions on multiple imputation. J. Clin. Epidemiol..

