# Supplementary material for: Distance and destination of retail meat alter multidrug resistant contamination in the United States food system
Source: Sci Rep. 2023 Nov 29;13:21024. doi: 10.1038/s41598-023-48197-z (PMC10687246; doi:10.1038/s41598-023-48197-z)
Supplement: Supplementary file 1 — Supplementary Tables. [file 41598_2023_48197_MOESM1_ESM.docx]

| **Table S1: Missing Data Analysis on Establishment Number Missingness: Beta-coefficient (95% CI)** | | | | |
| --- | --- | --- | --- | --- |
| **Variable** | **Matched vs. NA** | **Matched vs. unmatched** | **Matched vs. unmatched and NA** | **Matched and unmatched vs. NA** |
| Growth status | 0.04 (-0.01, 0.10) | **-0.12 (-0.16, 0.13)** | 0.04 (-0.01, 0.09) | 0.05 (-0.03, 0.10) |
| Meat type | **0.51 (0.49, 0.53)** | **0.48 (0.42, 0.54)** | **0.50 (0.48, 0.52)** | **0.49 (0.47, 0.51)** |
| State where purchased | -0.02 (-0.22, -0.01) | 0.05 (0.03, 0.06) | -0.01 (-0.02, -0.01) | -0.02 (-0.02, -0.01) |
| Organic status | **-1.15 (-1.33, -0.96)** | **-0.18 (-0.61, 0.26)** | **-1.07 (-1.24, -0.90)** | **-1.15 (-1.34, -0.97)** |
| Store where purchased | 0.00 (0.00, 0.00) | 0.00 (0.00, 0.00) | 0.00 (0.00, 0.00) | 0.00 (0.00, 0.00) |
| Brand of meat | 0.00 (0.00, 0.00) | 0.00 (0.00, 0.00) | 0.00 (0.00, 0.00) | 0.00 (0.00, 0.004) |
| Year purchased | **-0.11 (-0.14, -0.09)** | **-0.50 (-0.58, -0.42)** | **-0.15 (-0.17, -0.12)** | -0.09 (-0.11, -0.07) |
| MDR status | **-1.36 (-1.57, -1.16)** | 0.06 (-0.28, 0.39) | **-1.13 (-1.31, -0.95)** | **-1.37 (-1.57, -1.16)** |

A sensitivity analysis was performed to determine missingness for processor codes in the NARMS dataset. All available variables specific to the meat sample were recruited. Four separate sub analyses were conducted to account for either a missing processor code on the retail meat package itself or unmatched processor codes with a processor location when the processor code was listed in NARMS. *P* < 0.05 were bolded. Source: United States Food and Drug Administration National Antimicrobial Resistance Monitoring System.

| Table S2: Log-Binomial Analysis of MDR Bacteria-Contaminated Retail Meat and Risk Factors, Years 2012-2014, complete case analysis vs. multiple imputation by chained equations estimates | | | | | |
| --- | --- | --- | --- | --- | --- |
| Variables | | Unadjusted Primary Outcome: Consumer Exposure to MDR Bacteria, PR (95% CI) | Unadjusted Primary Outcome: Multiple Imputation Generated Results, PR (95% CI) | Adjusted Primary Outcome, aPR (95% CI) | Adjusted Primary Outcome: Multiple Imputation Generated Results, aPR (95% CI) |
| Year | |  |  |  |  |
|  | 2012 | REF^†^ | REF | - | - |
|  | 2013 | 0.78 (0.67-0.91)** | 0.82 (0.71-0.95)** | - | - |
|  | 2014 | 0.59 (0.50-0.70)*** | 0.65 (0.56-0.76)*** | - | - |
| Meat type | |  |  |  |  |
|  | Chicken Breast | REF | REF | - | - |
|  | Ground Turkey | 2.25 (1.94-2.61)*** | 2.30 (2.00-2.64)*** | - | - |
|  | Ground Beef | 0.21 (0.14-0.31)*** | 0.16 (0.12-0.22)*** | - | - |
|  | Pork Chop | 0.29 (0.19-0.45)*** | 0.24 (0.18-0.31)*** | - | - |
| State Sampled | |  |  |  |  |
|  | Georgia | REF | REF | REF | REF |
|  | Oregon | 1.04 (0.86-1.25) | 0.96 (0.80-1.15) | 0.93 (0.79-1.10) | 0.92 (0.78-1.08) |
|  | Minnesota | 0.07 (0.04-0.12)*** | 0.07 (0.04-0.11)*** | 0.06 (0.2-0.12)*** | 0.06 (0.04-0.11)*** |
|  | New York | 0.28 (0.20-0.38)*** | 0.24 (0.16-0.33)*** | 0.24 (0.18-0.33)*** | 0.23 (0.17-0.31)*** |
|  | Pennsylvania | 0.17 (0.12-0.25)*** | 0.15 (0.10-0.21)*** | 0.15 (0.10-0.22)*** | 0.14 (0.10-0.20)*** |
|  | Colorado | 0.05 (0.012-0.10)*** | 0.04 (0.02-0.08)*** | 0.05 (0.02-0.10)*** | 0.04 (0.02-0.08)*** |
|  | Tennessee | 0.92 (0.76-1.12) | 0.81 (0.67-0.98)* | 0.83 (0.69-1.00) | 0.81 (0.68-0.96)* |
|  | Maryland | 1.24 (1.04-1.48)* | 1.14 (0.96-1.35) | 1.12 (0.95-1.32) | 1.11 (0.95-1.29) |
|  | Washington | 0.04 (0.01-0.09)*** | 0.03 (0.01-0.07)*** | 0.03 (0.01-0.08)*** | 0.03 (0.01-0.07)*** |
|  | New Mexico | 0.09 (0.05-0.16)*** | 0.09 (0.05-0.14)*** | 0.08 (0.05-0.15)*** | 0.08 (0.05-0.14)*** |
|  | California | 0.03 (0.01-0.08)*** | 0.03 (0.02-0.07)*** | 0.02 (0.01-0.07)*** | 0.03 (0.02-0.07)*** |
|  | Missouri | 0.03 (0.01-0.09)*** | 0.03 (0.01-0.07)*** | 0.03 (0.01-0.08)*** | 0.03 (0.01-0.07)*** |
|  | Louisiana | 0.05 (0.02-0.12)*** | 0.04 (0.02-0.09)*** | 0.05 (0.02-0.12)*** | 0.04 (0.02-0.10)*** |
|  | Connecticut | 0.19 (0.12-0.30)*** | 0.15 (0.10-0.22)*** | 0.05 (0.02-0.10)*** | 0.14 (0.09-0.20)*** |
| Region Processed | |  |  |  |  |
|  | South | REF | REF | REF | REF |
|  | Midwest | 0.86 (0.74-1.00)* | 0.86 (0.74-1.00)* | 0.68 (0.58-0.79)*** | 0.68 (0.58-0.79)*** |
|  | West | 0.39 (0.31-0.50)*** | 0.39 (0.46-0.76 | 0.74 (0.58-0.94)* | 0.74 (0.58-0.95)* |
|  | Northeast | 0.59 (0.46-0.76)*** | 0.59 (0.46-0.76)*** | 0.67 (0.52-0.86)** | 0.67 (0.52-0.86)** |
| Distance Traveled Quartiles, (mi) | |  |  |  |  |
|  | 0-194 | REF^†^ | REF | REF^†^ | REF |
|  | 194-469 | 1.71 (1.40-2.10)*** | 1.80 (1.44-2.24)*** | 1.59 (1.31-1.94)*** | 1.78 (1.45-2.18)*** |
|  | 469-910 | 1.69 (1.38-2.07)*** | 1.59 (1.29-1.96)*** | 1.09 (1.89-1.34) | 1.24 (1.01-1.52)* |
|  | 910-2948 | 1.54 (1.26-1.88)*** | 1.54 (1.25-1.89)*** | 0.85 (0.69.-1.03) | 0.96 (0.79-1.17) |

Difference between complete case analysis and multiple imputed dataset analyses to investigate risk factors for MDR bacteria prevalence in retail meatPrevalence of overall bacteria and MDR bacteria contaminated retail meat was calculated by dividing the number of total or MDR bacteria contaminated samples over the total meat samples. Unadjusted models were evaluated to determine prevalence of MDR bacteria among all meat samples (human exposure) and MDR bacteria among contaminated meat samples (resister). Source: United States Food and Drug Administration National Antimicrobial Resistance Monitoring System.

*REF* referent group, *PR* Prevalence Ratio, *CI* Confidence Intervals, *aPR* Adjusted Prevalence Ratio.

**P*  < 0.05, ***P* < 0.01, ****P* < 0.001, † *P*-trend < 0.05

| Table S3: MDR Bacteria Among All Collected Retail Meat within Bacteria Genus. | | | | | | | | | | |
| --- | --- | --- | --- | --- | --- | --- | --- | --- | --- | --- |
|  | |  | *Salmonella* | | *Campylobacter* | | *E. coli* | | *Enterococcus* | |
| Risk Factor | | Meat Samples, n | MDR, n | Prevalence, % | MDR, n | Prevalence, % | MDR, n | Prevalence, % | MDR, n | Prevalence, % |
| Year | |  |  |  |  |  |  |  |  |  |
|  | 2012 | 3,125 | 69 | 2.21 | 6 | 0.19 | 215 | 6.88 | 11 | 0.35 |
|  | 2013 | 3,987 | 71 | 1.78 | 4 | 0.10 | 220 | 5.51 | 4 | 0.10 |
|  | 2014 | 4,131 | 26 | 0.63 | 3 | 0.07 | 196 | 4.74 | 10 | 0.24 |
| Meat type | |  |  |  |  |  |  |  |  |  |
|  | Chicken Breast | 3,736 | 87 | 2.33 | 13 | 0.35 | 111 | 2.97 | 14 | 0.37 |
|  | Ground Turkey | 4,152 | 69 | 1.66 | 0 | 0.00 | 483 | 11.63 | 10 | 0.24 |
|  | Ground Beef | 2,164 | 8 | 0.37 | 0 | 0.00 | 18 | 0.83 | 1 | 0.05 |
|  | Pork Chop | 1,191 | 2 | 0.17 | 0 | 0.00 | 19 | 1.59 | 0 | 0.00 |
| State Sampled | |  |  |  |  |  |  |  |  |  |
|  | Georgia | 1,083 | 8 | 0.74 | 1 | 0.09 | 183 | 16.90 | 4 | 0.37 |
|  | Oregon | 953 | 5 | 0.52 | 0 | 0.00 | 170 | 17.84 | 4 | 0.42 |
|  | Minnesota | 906 | 11 | 1.21 | 0 | 0.00 | - | - | - | - |
|  | New York | 889 | 43 | 4.84 | 2 | 0.22 | - | - | - | - |
|  | Pennsylvania | 868 | 19 | 2.18 | 8 | 0.92 | - | - | - | - |
|  | Colorado | 852 | 7 | 0.82 | 0 | 0.00 | - | - | - | - |
|  | Tennessee | 833 | 3 | 0.36 | 0 | 0.00 | 132 | 15.83 | 4 | 0.48 |
|  | Maryland | 810 | 21 | 2.59 | 2 | 0.25 | 146 | 18.00 | 13 | 1.60 |
|  | Washington | 776 | 5 | 0.64 | 0 | 0.00 | - | - | - | - |
|  | New Mexico | 735 | 12 | 1.63 | 0 | 0.00 | - | - | - | - |
|  | California | 721 | 4 | 0.55 | 0 | 0.00 | - | - | - | - |
|  | Missouri | 670 | 4 | 0.59 | 0 | 0.00 | - | - | - | - |
|  | Louisiana | 610 | 6 | 0.98 | 0 | 0.00 | - | - | - | - |
|  | Connecticut | 537 | 18 | 3.35 | 0 | 0.00 | - | - | - | - |
| Region Processed | |  |  |  |  |  |  |  |  |  |
|  | South | 4,976 | 118 | 2.37 | 13 | 0.26 | 307 | 6.17 | 19 | 0.38 |
|  | Midwest | 3,048 | 28 | 0.92 | 0 | 0.00 | 210 | 6.89 | 3 | 0.10 |
|  | West | 2,054 | 9 | 0.44 | 0 | 0.00 | 62 | 3.00 | 3 | 0.15 |
|  | Northeast | 1,165 | 11 | 0.94 | 0 | 0.00 | 52 | 4.46 | 0 | 0.00 |
| Distance Traveled quartiles (mi) | |  |  |  |  |  |  |  |  |  |
|  | 0-194 | 2,867 | 34 | 1.21 | 3 | 0.11 | 98 | 3.49 | 9 | 0.32 |
|  | 194-468 | 2,604 | 63 | 2.23 | 8 | 0.28 | 149 | 5.28 | 4 | 0.14 |
|  | 469-909 | 2,769 | 37 | 1.33 | 1 | 0.04 | 191 | 6.84 | 6 | 0.21 |
|  | 910-2948 | 3,003 | 32 | 1.14 | 1 | 0.04 | 193 | 6.88 | 6 | 0.21 |

Prevalence of MDR bacteria contaminated retail meat among all retail meat stratified by bacteria genus. Within each stratum, risk factors were investigated further. Dash lines indicate bacteria could not be cultured. Source: United States Food and Drug Administration National Antimicrobial Resistance Monitoring System.

| **Table S4: Number of meat samples shipped from processor region (by distance quartile), 2012-2014** | | | | |
| --- | --- | --- | --- | --- |
| Processor Region | Q1 (0-193mi) | Q2 (194-468mi) | Q3 (469-909mi) | Q4 (910-2948mi) |
| Midwest | 559 | 483 | 593 | 1,513 |
| Northeast | 443 | 410 | 133 | 179 |
| South | 844 | 1,573 | 1,674 | 885 |
| West | 1,021 | 238 | 369 | 426 |

Distance shipped from processor location to final store location was binned into quartiles. This table shows quartile distance by region where the meat was processed. Meat processed in the west had the highest proportion of short distances between processor and store location, whereas processors in the Midwest had the highest proportion of meat that traveled the furthest distances. Source: United States Food and Drug Administration National Antimicrobial Resistance Monitoring System.

| Table S5: Shipping Distances by Risk Factors | | | | | |
| --- | --- | --- | --- | --- | --- |
| Risk Factor | | Meat Samples, n | Minimum Distance Traveled (mi) | Maximum Distance Traveled (mi) | Median Distance Traveled (mi) |
| Year | |  |  |  |  |
|  | 2012 | 3,125 | 5.1 | 2858.2 | 445.5 |
|  | 2013 | 3,987 | 1.5 | 2806.0 | 472.9 |
|  | 2014 | 4,131 | 2.7 | 2948.4 | 485.6 |
| Meat type | |  |  |  |  |
|  | Chicken Breast | 3,736 | 1.8 | 2858.2 | 365.7 |
|  | Ground Turkey | 4,152 | 19.9 | 2738.2 | 691.9 |
|  | Ground Beef | 2,164 | 2.7 | 2948.4 | 290.3 |
|  | Pork Chop | 1,191 | 1.5 | 2806.0 | 262.4 |
| State Sampled | |  |  |  |  |
|  | Georgia | 1,083 | 40.2 | 1217.1 | 309.9 |
|  | Oregon | 953 | 5.5 | 2733.0 | 770.0 |
|  | Minnesota | 906 | 2.7 | 2049.0 | 174.5 |
|  | New York | 889 | 127.8 | 2948.4 | 489.0 |
|  | Pennsylvania | 868 | 22.0 | 2858.2 | 369.1 |
|  | Colorado | 852 | 1.8 | 1841.4 | 924.3 |
|  | Tennessee | 833 | 1.5 | 1333.4 | 528.6 |
|  | Maryland | 810 | 56.6 | 2719.0 | 238.6 |
|  | Washington | 776 | 20.9 | 2806.0 | 860.0 |
|  | New Mexico | 735 | 291.7 | 1846.5 | 918.3 |
|  | California | 721 | 1.8 | 1841.4 | 924.3 |
|  | Missouri | 670 | 22.9 | 2049.2 | 367.9 |
|  | Louisiana | 610 | 70.4 | 2539.6 | 626.7 |
|  | Connecticut | 537 | 103.2 | 1633.2 | 397.7 |
| Region Processed | |  |  |  |  |
|  | South | 4,976 | 1.5 | 2369.0 | 464.9 |
|  | Midwest | 3,048 | 2.7 | 2364.6 | 839.5 |
|  | West | 2,054 | 1.8 | 2948.4 | 200.3 |
|  | Northeast | 1,165 | 24.0 | 2806.0 | 230.7 |
| Distance Traveled quartiles (mi) | |  |  |  |  |
|  | 0-194 | 2,867 | 1.5 | 194.7 | 88.0 |
|  | 194-469 | 2,604 | 194.8 | 468.8 | 315.6 |
|  | 469-910 | 2,769 | 468.9 | 910.0 | 648.3 |
|  | 910-2948 | 3,003 | 910.0 | 2948.4 | 1229.6 |

| Table S6: MDR Bacteria-Contaminated Retail Meat Risk Factors within Bacteria Genus. | | | | | | | | | | | | | |
| --- | --- | --- | --- | --- | --- | --- | --- | --- | --- | --- | --- | --- | --- |
|  | | *Salmonella* | | | *Campylobacter* | | | *E. coli* | | | Enterococcus | | |
| Risk Factor | | Culturable Samples, n | MDR, n | Prevalence, % | Culturable Samples, n | MDR, N | Prevalence, % | Culturable Samples, n | MDR, n | Prevalence, % | Culturable Samples, n | MDR, n | Prevalence, % |
| Year | |  |  |  |  |  |  |  |  |  |  |  |  |
|  | 2012 | 261 | 82 | 31.42 | 457 | 6 | 1.31 | 715 | 264 | 36.92 | 884 | 12 | 1.36 |
|  | 2013 | 256 | 89 | 34.77 | 475 | 4 | 0.84 | 695 | 255 | 36.69 | 984 | 6 | 0.61 |
|  | 2014 | 212 | 34 | 16.04 | 374 | 4 | 1.07 | 666 | 215 | 32.28 | 1,024 | 11 | 1.07 |
| Meat type | |  |  |  |  |  |  |  |  |  |  |  |  |
|  | Chicken Breast | 1,426 | 480 | 33.66 | 4,754 | 31 | 0.65 | 2,332 | 748 | 32.08 | 2,888 | 62 | 2.15 |
|  | Ground Turkey | 1,454 | 510 | 35.08 | 128 | 4 | 3.13 | 3,252 | 1,992 | 61.25 | 2,047 | 56 | 2.74 |
|  | Ground Beef | 128 | 33 | 25.78 | 2 | 0 | 0.00 | 1,897 | 200 | 10.54 | 3,564 | 11 | 0.31 |
|  | Pork Chop | 138 | 38 | 27.54 | 12 | 0 | 0.00 | 1,431 | 361 | 25.23 | 3,665 | 6 | 0.16 |
| state Sampled | |  |  |  |  |  |  |  |  |  |  |  |  |
|  | Georgia | 207 | 91 | 43.96 | 301 | 5 | 1.66 | 3,027 | 1,030 | 34.03 | 3,176 | 11 | 0.35 |
|  | Oregon | 98 | 31 | 31.63 | 185 | 0 | 0.00 | 2,209 | 859 | 38.89 | 3,417 | 23 | 0.67 |
|  | Minnesota | 368 | 87 | 23.64 | 451 | 0 | 0.00 | 0 | 0 | - | 0 | 0 | - |
|  | New York | 502 | 241 | 48.01 | 543 | 3 | 0.55 | 0 | 0 | - | 0 | 0 | - |
|  | Pennsylvania | 241 | 69 | 28.63 | 237 | 8 | 3.38 | 0 | 0 | - | 0 | 0 | - |
|  | Colorado | 262 | 63 | 24.05 | 638 | 3 | 0.47 | 0 | 0 | - | 0 | 0 | - |
|  | Tennessee | 113 | 33 | 29.20 | 289 | 5 | 1.73 | 2,436 | 916 | 37.60 | 3,420 | 29 | 0.85 |
|  | Maryland | 332 | 144 | 43.37 | 238 | 3 | 1.26 | 1,888 | 705 | 37.34 | 2,561 | 77 | 3.01 |
|  | Washington | 39 | 8 | 20.51 | 117 | 0 | 0.00 | 0 | 0 | - | 0 | 0 | - |
|  | New Mexico | 559 | 149 | 26.65 | 504 | 2 | 0.40 | 0 | 0 | - | 0 | 0 | - |
|  | California | 304 | 42 | 13.82 | 877 | 4 | 0.46 | 0 | 0 | - | 0 | 0 | - |
|  | Missouri | 49 | 5 | 10.20 | 166 | 0 | 0.00 | 0 | 0 | - | 0 | 0 | - |
|  | Louisiana | 34 | 7 | 20.59 | 189 | 1 | 0.53 | 0 | 0 | - | 0 | 0 | - |
|  | Connecticut | 377 | 116 | 30.77 | 639 | 4 | 0.63 | 0 | 0 | - | 0 | 0 | - |
| Region Processed | |  |  |  |  |  |  |  |  |  |  |  |  |
|  | South | 332 | 120 | 36.14 | 624 | 13 | 2.08 | 830 | 304 | 36.63 | 908 | 20 | 2.20 |
|  | Midwest | 118 | 27 | 22.88 | 100 | 0 | 0.00 | 371 | 211 | 56.87 | 292 | 3 | 1.03 |
|  | West | 65 | 9 | 13.85 | 250 | 0 | 0.00 | 179 | 63 | 35.20 | 277 | 2 | 0.72 |
|  | Northeast | 44 | 10 | 22.73 | 27 | 0 | 0.00 | 162 | 53 | 32.72 | 231 | 0 | 0.00 |
| Distance Traveled quartiles (mi) | |  |  |  |  |  |  |  |  |  |  |  |  |
|  | 0-194 | 113 | 32 | 28.32 | 317 | 3 | 0.95 | 309 | 100 | 32.36 | 491 | 9 | 1.83 |
|  | 194-469 | 170 | 63 | 37.06 | 240 | 8 | 3.33 | 467 | 149 | 31.91 | 554 | 4 | 0.72 |
|  | 469-910 | 138 | 37 | 26.81 | 236 | 1 | 0.42 | 421 | 190 | 45.13 | 342 | 7 | 2.05 |
|  | 910-2948 | 137 | 34 | 24.82 | 207 | 1 | 0.48 | 343 | 192 | 55.98 | 320 | 5 | 1.56 |

Prevalence of MDR bacteria contaminated retail meat among contaminated retail meat stratified by bacteria genus. Within each stratum, risk factors were investigated further. Dash lines indicate bacteria could not be cultured.

| Table S7: MDR Bacteria-Contaminated Retail Meat Risk Factors within Meat Type. | | | | | | | | | | | | | |
| --- | --- | --- | --- | --- | --- | --- | --- | --- | --- | --- | --- | --- | --- |
|  | | Chicken Breast | | | Ground Turkey | | | Ground Beef | | | Pork Chop | | |
| Risk Factor | | Meat Samples, n | MDR Bacteria, n | Prevalence, % | Meat Samples, n | MDR Bacteria, n | Prevalence, % | Meat Samples, n | MDR Bacteria, n | Prevalence, % | Meat Samples, n | MDR Bacteria, N | Prevalence, % |
| Year | |  |  |  |  |  |  |  |  |  |  |  |  |
|  | 2012 | 768 | 89 | 0.12 | 489 | 197 | 0.40 | 194 | 7 | 0.04 | 131 | 8 | 0.06 |
|  | 2013 | 775 | 86 | 0.11 | 508 | 194 | 0.38 | 232 | 10 | 0.04 | 127 | 9 | 0.07 |
|  | 2014 | 669 | 50 | 0.07 | 484 | 171 | 0.35 | 248 | 10 | 0.04 | 166 | 4 | 0.02 |
| state Sampled | |  |  |  |  |  |  |  |  |  |  |  |  |
|  | Georgia | 342 | 27 | 0.08 | 356 | 157 | 0.44 | 196 | 3 | 0.02 | 184 | 9 | 0.05 |
|  | Oregon | 274 | 51 | 0.19 | 341 | 116 | 0.34 | 189 | 7 | 0.04 | 65 | 5 | 0.08 |
|  | Minnesota | 91 | 4 | 0.04 | 23 | 6 | 0.26 | 3 | 1 | 0.33 | 1 | 0 | 0.00 |
|  | New York | 132 | 38 | 0.29 | 20 | 5 | 0.25 | 1 | 1 | 1.00 | 2 | 1 | 0.50 |
|  | Pennsylvania | 120 | 21 | 0.18 | 26 | 5 | 0.19 | 2 | 1 | 0.50 | 2 | 0 | 0.00 |
|  | Colorado | 110 | 0 | 0.00 | 17 | 6 | 0.35 | 3 | 1 | 0.33 | 0 | 0 |  |
|  | Tennessee | 256 | 22 | 0.09 | 310 | 112 | 0.36 | 132 | 1 | 0.01 | 100 | 4 | 0.04 |
|  | Maryland | 298 | 48 | 0.16 | 286 | 123 | 0.43 | 140 | 9 | 0.06 | 66 | 2 | 0.03 |
|  | Washington | 89 | 1 | 0.01 | 10 | 3 | 0.30 | 1 | 1 | 1.00 | 3 | 0 | 0.00 |
|  | New Mexico | 113 | 1 | 0.01 | 27 | 10 | 0.37 | 3 | 1 | 0.33 | 1 | 0 | 0.00 |
|  | California | 121 | 2 | 0.02 | 14 | 2 | 0.14 | 1 | 0 | 0.00 | 0 | 0 | - |
|  | Missouri | 104 | 0 | 0.00 | 16 | 3 | 0.19 | 1 | 1 | 1.00 | 0 | 0 | - |
|  | Louisiana | 104 | 0 | 0.00 | 12 | 6 | 0.50 | 0 | 0 | - | 0 | 0 | - |
|  | Connecticut | 58 | 10 | 0.17 | 23 | 8 | 0.35 | 2 | 0 | 0.00 | 0 | 0 | - |
| Region Processed | |  |  |  |  |  |  |  |  |  |  |  |  |
|  | South | 1,515 | 168 | 0.11 | 587 | 266 | 0.45 | 266 | 4 | 0.02 | 318 | 19 | 0.06 |
|  | Midwest | 147 | 1 | 0.01 | 621 | 235 | 0.38 | 102 | 4 | 0.04 | 6 | 1 | 0.17 |
|  | West | 481 | 52 | 0.11 | 64 | 11 | 0.17 | 175 | 11 | 0.06 | 45 | 0 | 0.00 |
|  | Northeast | 69 | 4 | 0.06 | 209 | 50 | 0.24 | 131 | 8 | 0.06 | 55 | 1 | 0.02 |
| Distance Traveled quartiles, (mi) | |  |  |  |  |  |  |  |  |  |  |  |  |
|  | 0-194 | 687 | 96 | 0.14 | 102 | 33 | 0.32 | 240 | 8 | 0.03 | 201 | 7 | 0.03 |
|  | 194-469 | 716 | 96 | 0.13 | 282 | 110 | 0.39 | 252 | 7 | 0.03 | 177 | 11 | 0.06 |
|  | 469-910 | 509 | 25 | 0.05 | 536 | 201 | 0.38 | 72 | 6 | 0.08 | 18 | 3 | 0.17 |
|  | 910-2948 | 300 | 8 | 0.03 | 561 | 218 | 0.39 | 110 | 6 | 0.05 | 28 | 0 | 0.00 |

Prevalence of MDR bacteria contaminated retail meat among all retail meat stratified by meat type. Within each stratum, risk factors were investigated further. Dash lines indicate bacteria could not be cultured.
